# Dual‐Responsive Chitosan‐Grafted PNIPAAm Hydrogel Eye Drop Incorporating Insulin‐Imprinted Microgels for Dry Eye Syndrome Treatment

**DOI:** 10.1002/mabi.202600003

**Published:** 2026-06-18

**Authors:** Masoumeh Alsadat Hosseini, Reihaneh Khademi, Mahshid Shokri, Reza Ghiasi, Ali Doostmohammadi, Mahshid Kharaziha, Sahar Salehi

**Affiliations:** ^1^ Department of Materials Engineering Isfahan University of Technology Isfahan Iran; ^2^ Department of Clinical Science Faculty of Veterinary Medicine Islamic Azad University Garmsar Iran; ^3^ Department of Mechanical Engineering Lassonde School of Engineering York University Toronto Canada; ^4^ Bioengineering Institute of Technology Universitat Internacional de Catalunya Barcelona Spain; ^5^ Department of Bioengineering Universitat Internacional de Catalunya Barcelona Spain; ^6^ Department of Biomaterials Faculty of Engineering Science University of Bayreuth Bayreuth Germany; ^7^ Department of Biointelligent System Engineering Institute of Food Science and Biotechnology University of Hohenheim Stuttgart Germany; ^8^ Fraunhofer Institute for Manufacturing Engineering and Automation (IPA) Stuttgart Germany

**Keywords:** dry eye syndrome, dual‐responsive, molecularly imprinted microgels, ocular drug delivery, Poly‐N‐isopropylacrylamide

## Abstract

Dry eye syndrome (DES) happens as a result of inadequate tear secretion or excessive evaporation, which leads to ocular surface irritation and damage. A major therapeutic challenge is maintaining effective drug concentrations, as topical formulations are rapidly cleared by blinking and tear turnover. To address this limitation, we developed a dual pH‐ and temperature‐responsive composite hydrogel composed of chitosan (Cs)‐grafted poly‐N‐isopropylacrylamide (PNIPAAm)‐ CPN integrated with alginate‐based insulin‐imprinted microgels (IMIP). Incorporation of IMIP particles allowed tuning of rheology, swelling behavior, wettability, and biodegradation while preserving proper topical administration. Microgel addition increased viscosity from 3.1 mPa·s (CPN‐IMIP10) to 37.3 mPa·s (CPN‐IMIP35), and the sol–gel transition between 25 and 37°C enabled flowability at room temperature followed by rapid gelation on the ocular surface. All CPN‐IMIP hydrogels exhibited elastic‐dominant behavior (G′ > G″), confirming formation of a stable, shear‐resistant network. Insulin release was tunable: at pH 6.4 and 25°C, diffusion governed delivery (29.8 ± 3.6% at 48 h), whereas at pH 7.4 and 37°C, swelling‐controlled release predominated (54.4 ± 4.8%). The composite hydrogels supported human corneal epithelial cell viability and enhanced tear secretion, preserved corneal integrity, and reduced inflammation in a rat DES model. This system provides a minimally invasive, long‐acting therapeutic platform for DES.

## Graphical abstract

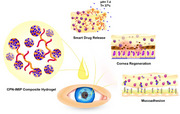

## 1 Introduction

Dry eye syndrome (DES) is a multifactorial disorder of the ocular surface that occurs when tear production is insufficient to maintain proper lubrication. It may lead to ocular inflammation and, in severe cases, impaired vision. This condition can result from a wide range of factors, including aging, environmental stress, some medications, and systemic diseases such as diabetes mellitus [1]. The main treatment for DES involves the topical application of artificial tears and immunomodulatory drugs such as Cyclosporine A (CsA), Epigallocatechin gallate (EGCG), Pimecrolimus (PMS), and Tacrolimus (also known as FK506) [2]. In recent years, insulin (INS) has attracted attention as a potential therapy. The presence of INS receptors on the ocular surface, particularly the corneal epithelium and lacrimal gland (LG), suggests that INS plays an important role in healing and maintenance of ocular surface integrity. Previous research has shown that INS can promote epithelial cell proliferation and accelerate corneal regeneration by supporting cellular metabolic activity and reducing inflammation [3]. In other words, artificial tear formulations act by lubricating the ocular surface, stabilizing the tear film, and diluting soluble inflammatory mediators to replicate the function of real human tears and promote ocular surface health [4, 5]. However, due to reflex tearing, blinking, and rapid anterior corneal changes, artificial tears might remain on the ocular surface for less than 5 min, which would limit their bioavailability [4].

To overcome the drawbacks of artificial tears, researchers often enhance the viscosity by using hydrogels [6, 7]. The 3D hydrogel network allows it to inflate quickly in water and hold a significant volume of water without being dissolved [8]. To increase the bioavailability, various environmentally sensitive hydrogels, including pH [9], temperature [10], and ion‐sensitive [11] ophthalmic hydrogels, have been created for ocular delivery. When the temperature varies, temperature‐sensitive hydrogels experience a reversible sol‐gel transition [12, 13]. Poly‐N‐isopropylacrylamide (PNIPAAm) is one of the frequently used temperature‐sensitive polymers, and its lower critical solution temperature (LCST) falls within the range of 30–35°C, which is close to the surface temperature of the human body [14]. However, the LCST of the PNIPAAm‐based systems highly depends on polymer composition and can vary from its typical value [15]. Numerous drug‐delivery hydrogels based on PNIPAAm have been developed to treat DES. For instance, Luo et al. [16] created a mucoadhesive drug delivery system based on PNIPAAm for the local treatment of DES. The resulting hydrogel showed temperature‐responsive behavior and allowed the EGCG molecules to be released over time. An EGCG‐loaded carrier was able to effectively repair corneal epithelial defects in a rabbit model while reducing cellular inflammation, oxidative stress, and apoptosis. However, cytotoxicity and non‐biodegradability are 2 challenges associated with PNIPAAm [17]. To overcome these drawbacks, grafting PNIPAAm onto natural polymers has been introduced as an efficient solution [18, 19]. Because of superior biocompatibility, low toxicity, and immune‐stimulating properties, chitosan (Cs) has been extensively used in drug delivery systems, particularly for ocular, nasal, oral, and transdermal applications. Grafting PNIPAAm onto Cs improves the overall biocompatibility and enzymatic biodegradability of the resulting copolymer. This enhanced biocompatibility is attributed to reducing free PNIPAAm chains and introducing bioactive amine groups. Therefore, it improves biological interactions and reduces inflammatory response [15].

Despite the promising potential of hydrogels for drug delivery to treat DES, challenges remain, particularly the burst release and limited absorption of specific therapeutic agents, such as hydrophobic drugs, proteins, and antibodies [20]. To overcome these issues, composite hydrogels incorporating nano and microcarriers such as liposomes, micelles, dendrimers, polymer‐based particles, and nanotubes have been developed [2, 21]. Such drug delivery systems improve drug absorption in hydrogels and prolong drug release. Because of the appropriate selectivity for target molecules and controlled drug release properties, molecularly imprinted polymers (MIPs) have gained considerable attention [22, 23]. In our previous study [24], we developed calcium alginate‐based MIP microgels for controlled insulin delivery. We found out that insulin release was slower under normal ocular conditions (pH 6.4) and significantly accelerated under dry eye conditions (pH 7.4). However, these microgels still faced the challenge of maintaining long‐term controlled release, which is particularly critical for the management of advanced DES.

To the best of our knowledge, the encapsulation of MIP within the hydrogel matrix has not been widely applied, especially for the treatment of DES. This study aims to develop a dual pH and temperature‐responsive ophthalmic hydrogel for the DES treatment. This composite hydrogel was based on Cs‐grafted‐PNIPAAm (Cs‐g‐PNIPAAm) as a matrix and encapsulated insulin‐imprinted microgels (IMIPs) based on calcium alginate as fillers. Moreover, the role of hydrogel composition on the physical and mechanical properties, as well as the adsorption and release of INS from composite hydrogels, was investigated in simulated tear fluid (STF) at 25°C and 37°C and at pH values mimicking healthy eye (6.4) and dry eye (7.4). Cytotoxicity assays conducted using human corneal epithelial cells (HCECs) over 7 days, together with *in vivo* transplantation in rats, confirmed the hydrogels' biocompatibility and therapeutic efficiency under physiological conditions. Considering these results, IMIP incorporated Cs‐g‐PNIPAAm hydrogels (CPN‐IMIP) could act as efficient and biocompatible drug delivery and tissue regeneration platforms for future biomedical applications.

## 2 Results and Discussion

### 2.1 Physicochemical Characterizations of Cs‐g‐PNIPAAm

**FIGURE 1:**
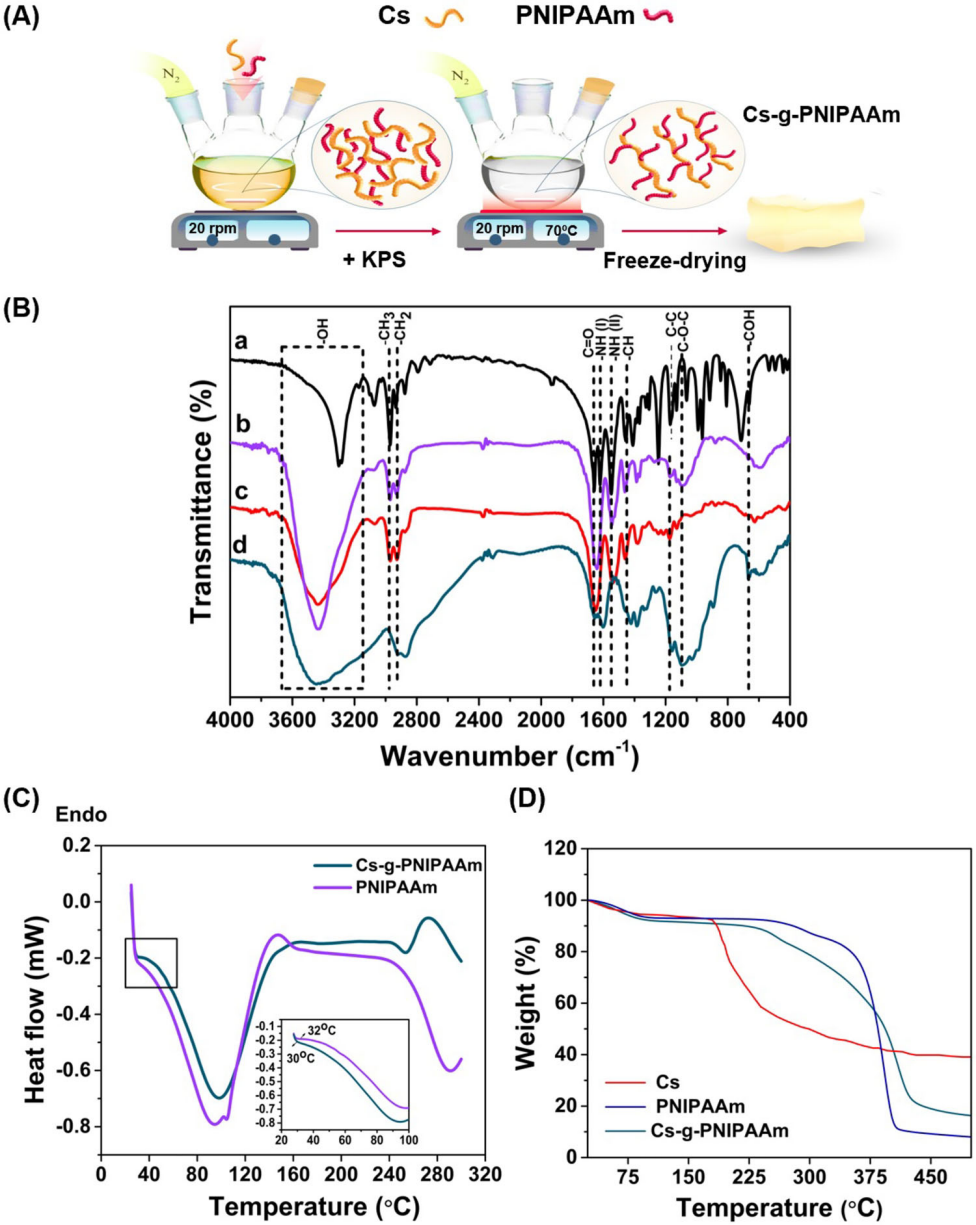
Physicochemical properties of Cs‐g‐PNIPAAm: (A) Schematic representing the copolymer synthesis process, (B) FTIR spectra of (a) NIPAAm, (b) Cs, (c) PNIPAAm, and (d) Cs‐g‐PNIPAAm, (C) DSC thermograms of PNIPAAm and Cs‐g‐PNIPAAm, and (D) TGA of Cs, PNIPAAm, and Cs‐g‐PNIPAAm.

A Cs‐g‐PNIPAAm copolymer was synthesized to serve as the matrix of the composite hydrogel. As shown in Figure 1A, the copolymer was prepared by free radical polymerization of Cs with NIPAAM in an aqueous medium, using potassium persulfate (KPS) as the initiator. Figure 1B illustrates the Fourier Transform Infrared (FTIR) spectrum of Cs‐g‐PNIPAAm, in comparison with those of Cs, NIPAAm, and PNIPAAm. The spectrum of Cs consisted of the characteristic absorption bands at 1659 and 1599 cm^−1^ attributed to the amide I and amide II bands, respectively. Additionally, the absorption bands at 1421 and 1138 cm^−1^ were referred to the C─H bending, while the signals at 1159 and 1074cm^−1^ were assigned to C─O─C and C─OH bonds of the anhydroglucose ring in the Cs structure, respectively [25]. The spectrum of NIPAAm consisted of the bands at 2970 cm^−1^ and 2936 cm^−1^ related to asymmetric C─H stretching vibrations of isopropyl and methylene groups [26]. Furthermore, the absorption bands at 1657 and 1620 cm^−1^ were assigned to the C═O stretching and N─H bending of the primary amide, while the identified band at 1547 cm^−1^ corresponded to secondary amide N─H deformation [27]. The 3 identified bands at 1460, 1387, and 1367 cm^−1^ corresponded to the dimethyl group vibrations, and the band at 1171 cm^−1^ was due to the C─C vibration in the isopropyl group of NIPAAm [28]. The spectra of PNIPAAm and Cs‐g‐PNIPAAm also consisted of distinctive bands corresponding to the isopropyl groups. The disappearance of the band at 1620 cm^−1^ following polymerization confirmed the cleavage of the C═C bond in PNIPAAm, confirming the formation of extended PNIPAAm chains. Strong absorption bands at 1651 and 1545 cm^−1^ were attributed to C═O stretching and N─H bending vibration of the secondary amide group [29]. Compared to the spectrum of PNIPAAm, Cs‐g‐PNIPAAm consisted of a new and strong absorption band at 1157 cm^−1^, which was attributed to the C─O─C stretching of the ether linkage in the CS backbone [27]. These findings confirmed the successful synthesis of the Cs‐g‐PNIPAAm copolymer. In this study, the grafting ratio, grafting efficiency, and total monomer conversion for the copolymer were estimated to be about 124%, 74%, and 60%, respectively, which are comparable to other studies [24]. The resultant copolymer was soluble in water, and at temperatures below the lower critical solution temperature (LCST), the final product had a translucent appearance, while above LCST, the copolymer solution became opaque and white [30]. Figure 1C shows the DSC curves of PNIPAAm and Cs‐g‐PNIPAAm copolymer.

The first endothermic peak of the DSC thermogram was used to determine the phase transition temperature of the samples, and the onset point of this peak was considered the LCST of the samples [31, 32]. An endothermic peak and an exothermic peak were observed in the DSC curve of Cs‐g‐PNIPAAm at around 100°C and 300°C, respectively. The endothermic peak corresponded to the glass transition temperature of NIPAAm, while the exothermic peak related to the disintegration of Cs. The presence of these 2 peaks supported the FTIR spectra and confirmed that the copolymer was composed of Cs and PNIPAAm [33, 34]. Moreover, it was found that the LCST of PNIPAAm was around 30°C, which was enhanced to 32°C at Cs‐g‐PNIPAAm copolymer (indicated in the inset of Figure 1C). The findings are consistent with the results obtained in earlier investigations [35, 36].

Due to the hydrophilic nature of Cs, its copolymerization with NIPAAm helped balance hydrophilic and hydrophobic interactions within the copolymer. This balance increased the hydrogen bonding with water molecules and consequently, the LCST of the copolymer, as reported in previous studies [37]. The TG analysis of Cs, PNIPAAm, and Cs‐g‐PNIPAAm copolymer is presented in Figure 1D. PNIPAAm and Cs showed one‐step degradation behavior. The TG curve for Cs consisted of a relatively low initial decomposition temperature (180°C), followed by a slower rate of degradation at higher temperatures. In contrast, the TG curve of PNIPAAm showed a much higher initial decomposition temperature (398°C). However, once the temperature was enhanced, rapid decomposition occurred. Two distinct degradation stages were observed in the Cs‐g‐PNIPAAm copolymer: the initial stage corresponded to Cs degradation, followed by the PNIPAAm chain degradation [37, 38]. These 2 distinct stages of degradation confirmed the existence of multiple phases in the structure and the successful copolymer formation. The TG curve of the Cs‐g‐PNIPAAm copolymer had a lower initial decomposition temperature than PNIPAAm, and a significant decomposition rate compared to Cs due to the presence of Cs in the structure [39]. A significant residual mass at 500°C for the Cs‐g‐PNIPAAm copolymer was measured. These findings confirmed the presence of Cs in the copolymer, as PNIPAAm was almost fully degraded at this temperature [33, 39].

### 2.2 Physicochemical Characterizations of IMIPs

**FIGURE 2:**
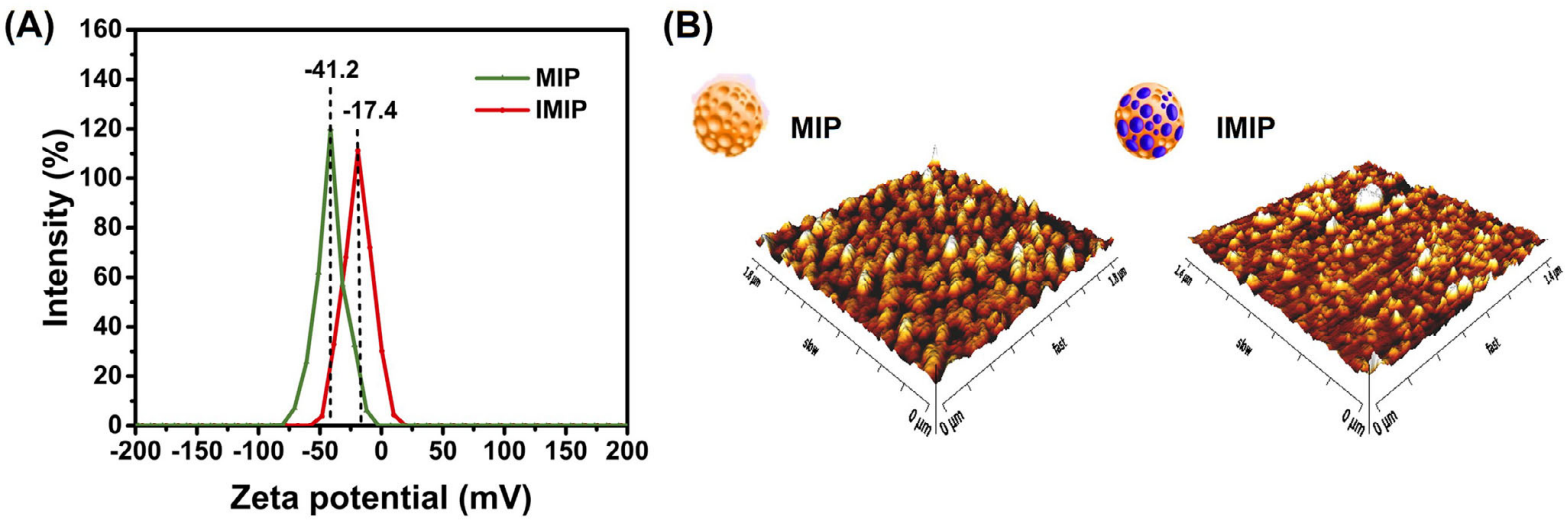
Characterization of IMIPs: (A) Zeta potential measurements of MIPs and IMIPs, and (B) AFM images of the surface of MIPs and IMIPs.

IMIPs were created before the synthesis of composite hydrogels. As reported in our previous research [24], the particle size of MIPs was measured to be 9–11 µm. Also, the zeta potential of MIPs and IMIPs in Figure 2A showed that both particles had good stability. A higher zeta potential value of IMIP than that of MIP might be attributed to the presence of available carboxylate groups in the adsorbed INS. Moreover, the AFM images in Figure 2B confirmed that IMIPs had lower surface roughness compared to MIPs, suggesting that INS was absorbed into the active binding sites on the surface. However, the presence of the template led to changes in the surface characteristics of microgels [24, 40].

### 2.3 Physicochemical Characterizations of Composite Hydrogels

**FIGURE 3:**
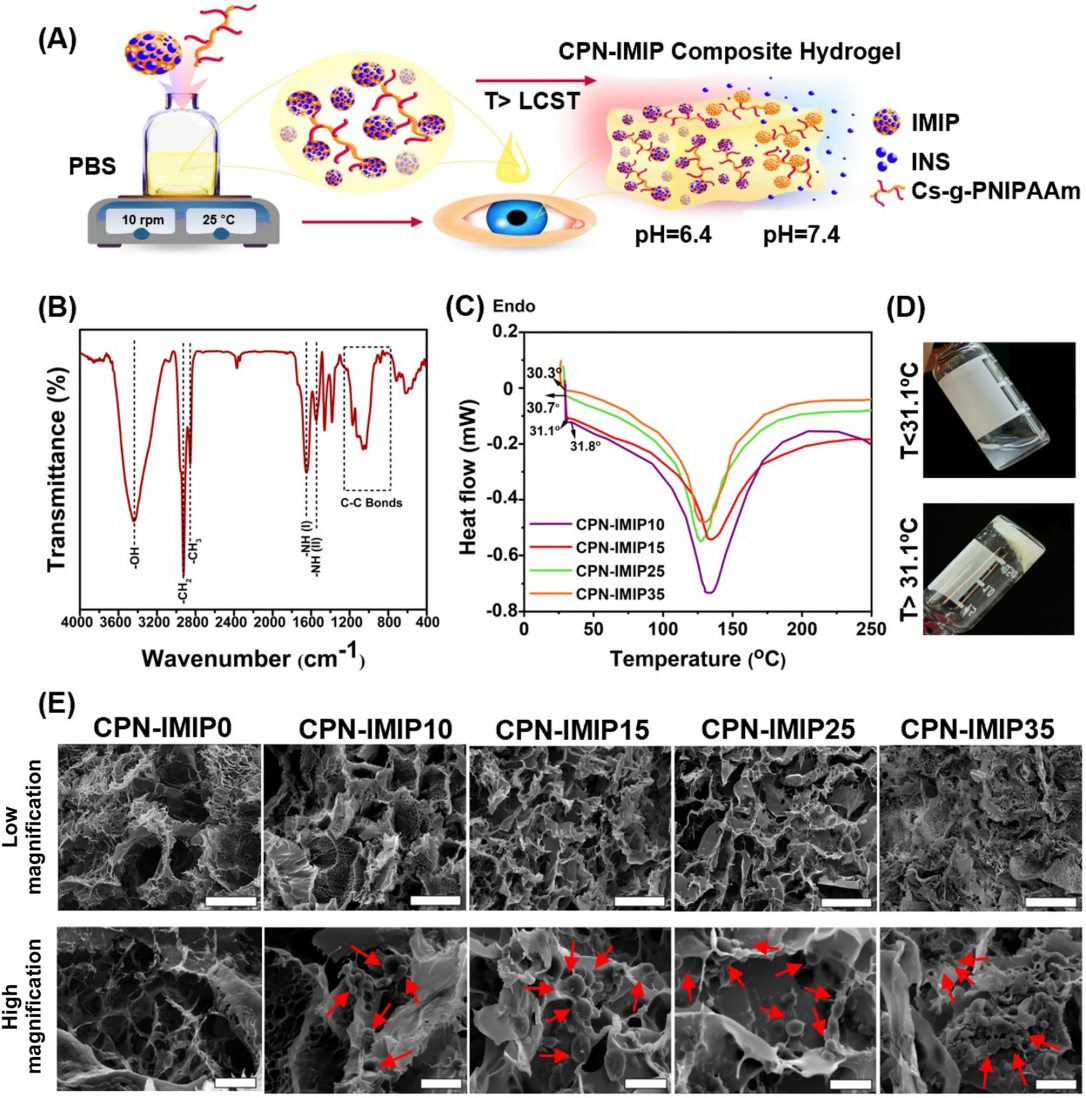
Physicochemical properties of CPN‐IMIP hydrogels: (A) Schematic representing the synthesis process and the presence of microgels within the composite hydrogel's matrix, (B) FTIR spectrum of CPN‐MIP15, (C) DSC thermograms of composite hydrogels, (D) thermoresponsive behavior of CPN‐IMIP15 above and below LCST, (E) cross‐sectional SEM images of composite hydrogels captured at 2 different magnifications (100 and 20 µm); red arrows show IMIPs within the matrix.

Next, composite hydrogels based on Cs‐g‐PNIPAAm and IMPs were developed for controlled and sustained INS release. A schematic of the synthesis and function of CPN‐IMIP composite hydrogel is presented in Figure 3A. As can be seen, after mixing IMIPs with Cs‐g‐PNIPAAm, applying it on the eye surface, which has a temperature higher than the hydrogel's LCST, results in the formation of a crosslinked structure. This resulting hydrogel can release the drug in response to pH and temperature changes. The FTIR spectrum of the hydrogel eye drop sample (CPN‐IMIP15) is presented in Figure 3B. Compared to the spectra of the copolymer in Figure 1B, the OH bond in the composite hydrogel showed an increased intensity. This could be due to the overlap of OH bonds within the IMIPs, Cs‐g‐PNIPAAm, and composite structure [41]. Moreover, the intensity of signals at 2970 and 2936 cm^−1^ related to asymmetric C─H stretching vibrations increased. This confirmed the improved hydrophobic bonding of IMIPs within the Cs‐g‐PNIPAAm matrix. This phenomenon led to the formation of a more compact structure. The intensity of the bands corresponding to the bending motions of amide I and amide II groups at about 1599 and 1531 cm^−1^ increased. This might be due to the formation of hydrogen bonds between the hydroxyl groups of alginate and the amino or hydroxyl groups of Cs [42, 43]. Moreover, the peak at 1157 cm^−^^1^, corresponding to the C─O─C stretching of the Cs ether ring, was slightly shifted to the left in the spectrum of the composite hydrogel. This shift suggested the modification in glycosidic bonds and the formation of new interactions between the Cs matrix and microgels [44]. According to these results, the formation of new characteristic peaks was not observed in the spectrum of the composite hydrogel. Therefore, IMIPs were physically incorporated within the hydrogel network only through hydrogen bonding and electrostatic interactions.

Since a gelling eye drop formulation should transform into a gel instantly upon contact with the eye to enhance bioavailability, a transition of a few seconds is desirable. The addition of microgels did not affect the transition kinetics, and all formulations exhibited a rapid sol‐to‐gel transition. However, the addition of microgels to hydrogels slightly reduced the LCST. The results of DSC for all hydrogel formulations and proof of the thermoresponsive behavior of CPN‐IMIP hydrogel, captured from the hydrogels in glass bottles, are presented in Figure 3C,D, respectively. For the CPN‐IMIP10 and CPN‐IMIP15 samples, the LCST values were 31.8°C and 31.1°C, respectively. Similarly, for the CPN‐IMIP25 and CPN‐IMIP35 samples, the LCST values decreased to 30.7°C and 30.3°C, respectively. The addition of IMIPs to the matrix hydrogel may lead to a decrease in LCST by disrupting hydrogen bond interactions, increasing hydrophilicity, and competitively binding with the PNIPAAm component. Similarly, Bellotti et al. [13] demonstrated that the inclusion of microparticles in hydrogels resulted in a reduction in the phase transition temperature. The sol‐to‐gel transition temperature of PNIPAAm hydrogel was reduced by incorporating varying concentrations of polylactic‐glycolic acid (PLGA) microparticles loaded with brimonidine tartrate. This modification enhanced the suitability of the hydrogel for lower‐than‐normal temperatures on the eye surface, such as during cold weather or wind exposure [13]. In addition, ophthalmic formulations are required to have acceptable pH values to ensure their safety and comfort. In this study, the pH of all formulations was also evaluated. pH values of CPN‐IMIP0 to CPN‐IMIP35 ranged from 6.3 ± 0.2 to 6.5 ± 0.5. Although these values were slightly acidic compared to physiological pH, they remained within an acceptable range for ophthalmic applications [45]. SEM images in Figure 3E showed that all hydrogels have a 3D porous and interconnected morphology, making them suitable platforms for cell growth and drug release [46]. The porous structure further facilitates nutrient transport and waste removal, thereby supporting cell proliferation [47, 48]. In addition, at higher microgel concentrations, such as CPN‐IMIP35, composite hydrogels displayed less uniform porosity, likely due to microgel aggregation within the polymeric network. The CPN‐IMIP15 sample showed the most uniform distribution of microgels and structural porosity compared to other samples. The red‐colored arrows, marked in the image, showed the presence of microgels distributed in hydrogel matrices. As reported in our previous research [24] and evident from SEM images, IMIPs have particle sizes less than 10 µm. A decreasing trend in the average pore size of the hydrogels after increasing the concentrations of microgels was also observed and measured in SEM images (81.8 ± 4.0 and 9.2 ± 3.1 µm for CPN‐IMIP0 and CPN‐IMIP35, respectively). The observed phenomenon could potentially be attributed to the presence of ionic interactions or crosslinks between alginate microgels and the hydrogel matrix, as described above, which might restrict the mobility of polymer chains and result in the creation of smaller pores within the hydrogel structure [49].

### 2.4 Transparency of Composite Hydrogels

**FIGURE 4:**
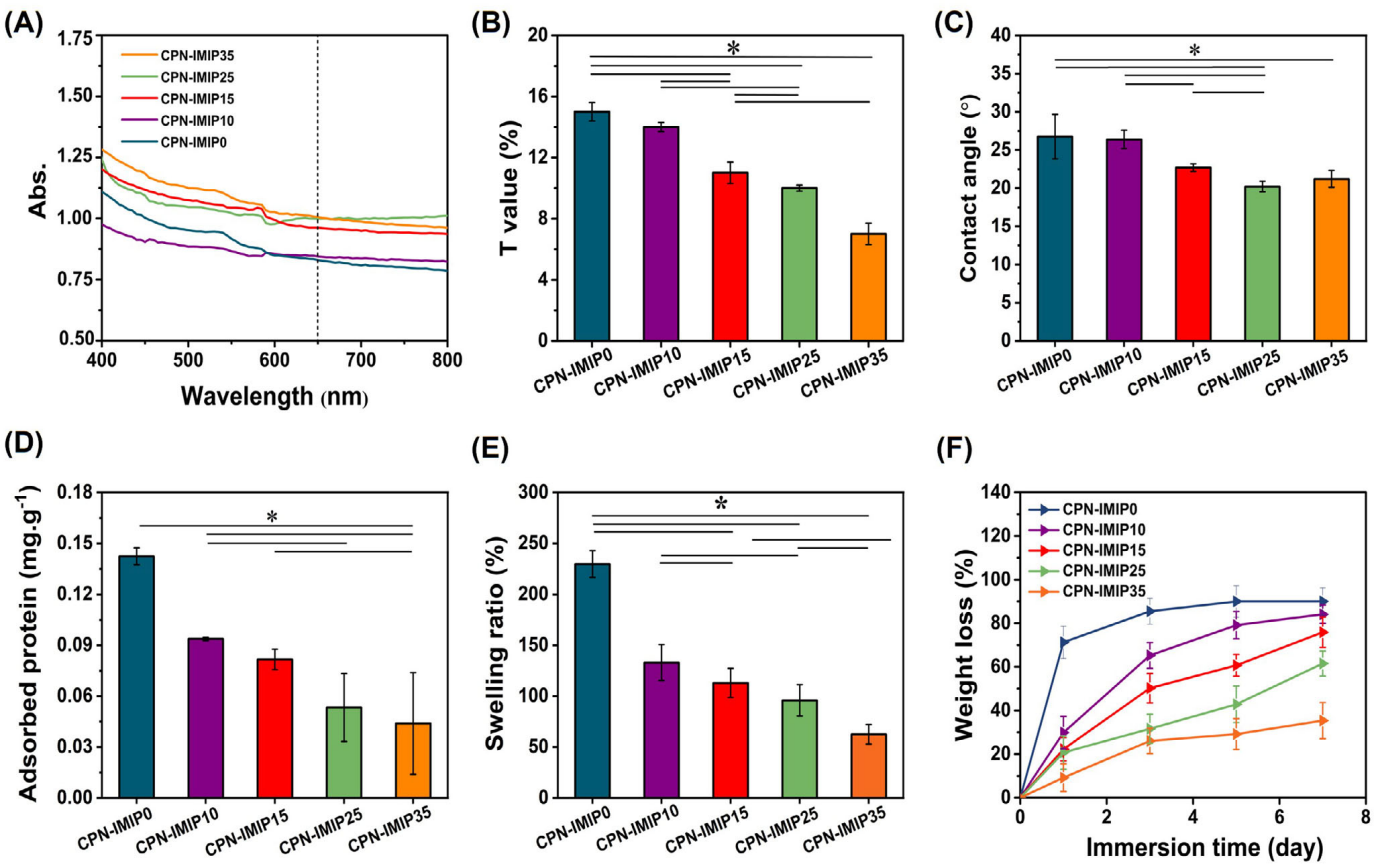
Physiological stability of CPN‐IMIP hydrogels: (A) UV‐Vis spectra, (B) T values of hydrogels, (C) water contact angles of hydrogels, (D) protein adsorption of hydrogels after 2 h incubation in BSA solution, (E) the swelling ratio (SR) of hydrogels after 1 h soaking in simulated tear fluid (STF), and (F) weight loss of hydrogels in STF (n = 3; *p < 0.05).

The transparency of hydrogels was further evaluated using UV‐Vis spectroscopy at room temperature. The UV‐Vis spectra and T values of hydrogel samples are presented in Figure 4A,B. It is evident that as the IMIP concentration in the composite increased, transparency decreased. Moreover, T values indicated that all samples except CPN‐IMIP35 were translucent. Since this formulation is designed to be used in small amounts as eye drops and forms a thin layer on the ocular surface, this level of transparency is unlikely to affect vision. Therefore, the optical behavior of composite hydrogels was dependent on the concentration of IMIPs and was expected to have proper optical properties for ocular application.

### 2.5 Physiological Stability of Composite Hydrogels

To investigate the *in vitro* wettability of the hydrogel eye drops, the water contact angle was determined, given its critical relationship with tear film dispersion and stability upon insertion [50]. As shown in Figure 4C, by increasing the MIP concentration up to 25 mg.mL^−1^, the contact angle values have decreased, indicating improved surface wettability. However, the water contact angle slightly increased for CPN‐IMIP35. At higher concentrations (i.e., 35 mg.mL^−1^), the aggregation of IMIPs led to the formation of larger clusters or uneven distribution on the hydrogel surface, leading to an increase in surface roughness or the creation of micro‐textures, which could trap air under a water droplet, decreasing the hydrophilicity of hydrogels [50, 51]. This effect partially counteracted the influence of the hydrophilic hydroxyl and carboxyl groups, which would otherwise enhance hydrophilicity. Next, we evaluated the protein adsorption of the composite hydrogels by incubating the materials in BSA solution for 2 h (Figure 4D). It is reported that over 400 distinct proteins make up the tear film, and the accumulation of the tear proteins in ocular hydrogels may raise the possibility of inflammation and microbial attachment [52]. It can be seen that protein adsorption from the BSA solution decreased with increasing IMIP content in the hydrogels and remained nearly constant for samples containing 25 and 35 mg.mL^−1^ IMIPs. This could be attributed to the hydrophilic nature of hydrogels, improved with increasing IMIP content to 25 mg.mL^−1^, as proved by measuring wettability. Hydrophilic surfaces typically resist protein adsorption more effectively than hydrophobic surfaces because proteins tend to adsorb more readily onto hydrophobic surfaces due to hydrophobic interactions [52, 53].

As the hydrophilicity of the surface was enhanced with increasing IMIP content (Figure 4C), the hydrogel may retain more water at the surface, forming a hydration layer. This hydration layer could act as a barrier to protein adsorption by preventing direct contact between the proteins and the hydrogel surface. In addition, the presence of INS associated with IMIPs might generate repulsive interactions with incoming proteins, which contribute to the reduced protein adsorption observed at higher IMIP concentrations [54]. Swelling ability is a critical factor in drug release, affecting kinetics and therapeutic efficacy. Controlled swelling of hydrogels in the physiological eye conditions ensures precise drug retention and distribution in the eye. Controlled swelling ability affects patient comfort and compliance, improving overall eye disease management [55]. Therefore, we measured the swelling ratio (SR) after incubating in STF at 37°C for 1 h (Figure 4E). The SR of the hydrogels showed a decreasing trend from 133.0 ± 17.5 to 62.5 ± 9.5% when the MIP concentration was increased from 10 to 35 mg.mL^−1^. This could be due to the presence of IMIPs within the hydrogel matrix that reduced the mobility of polymer chains and thus affected the pore sizes of hydrogels. Moreover, the higher number of microgels within the same hydrogel volume might reduce the available space for water uptake, therefore lowering the overall swelling capacity [56]. In a study by Prabhu et al. [57], various hydrogels were synthesized for ocular delivery of gentamicin and dexamethasone. These hydrogels had SRs ranging from 22.5% to 365.56% and were considered to be in a proper range for an ocular delivery system [57]. Therefore, in this study, based on the measured SRs of all compositions, they all stand in an appropriate range to be applied in the intraocular environment.

We also investigated the physiological stability of composite hydrogels in STF over 7 days. According to Figure 4F, the CPN‐MIP10 exhibited rapid degradation (72 ± 1% weight loss in 3 days and 93 ± 3% in 7 days) and almost the same behavior as the degradation behavior of the pure sample (93 ± 2% weight loss in 7 days). On the other hand, the CPN‐IMIP15 showed a weight loss of 55.8 ± 2.7% after 3 days of incubation. These results indicate that higher IMIP content slows down the degradation rate, which can be attributed to a denser network of the composite hydrogel and a reduced SR (Figure 4E) [58].

### 2.6 Rheological Properties of Composite Hydrogels

**FIGURE 5:**
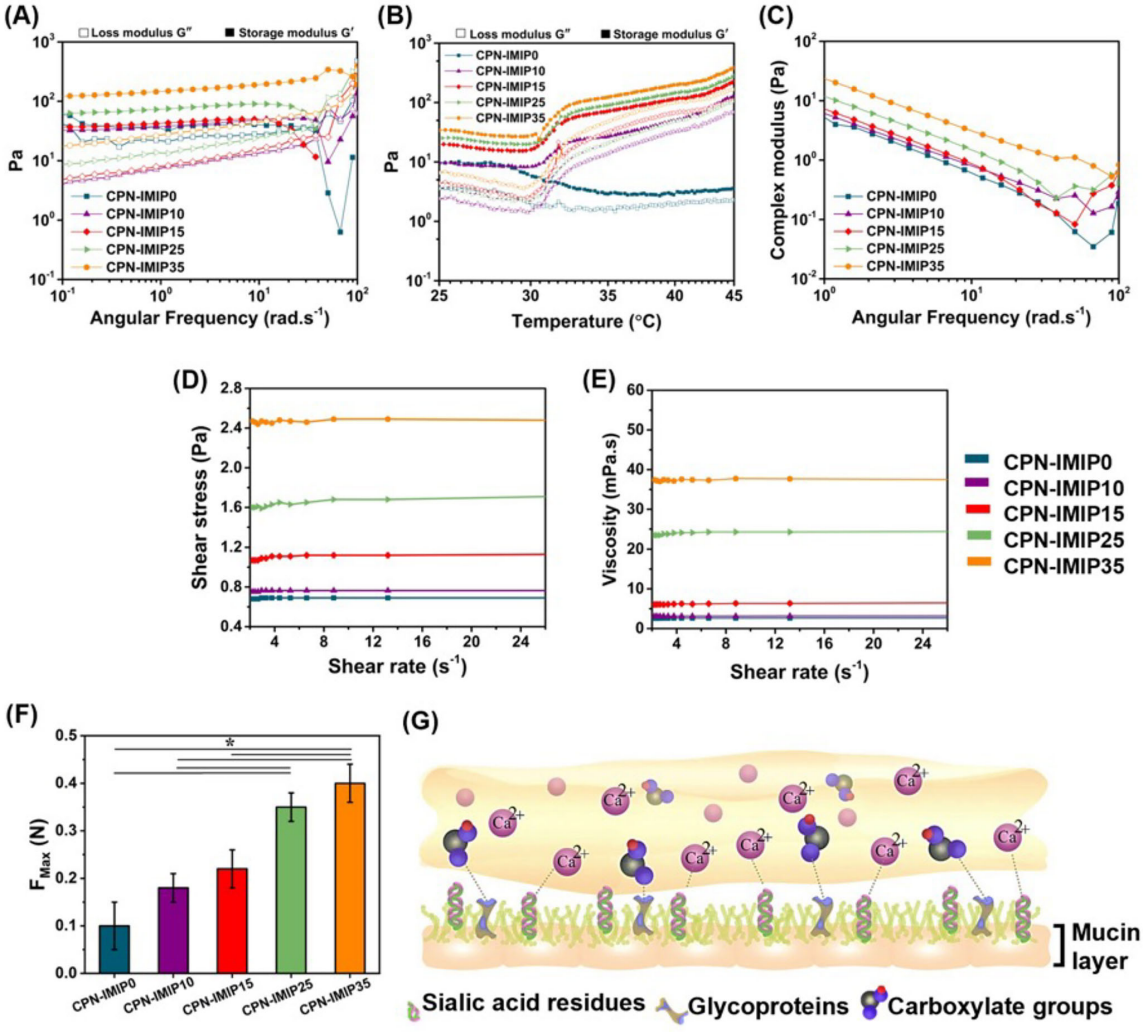
The rheological properties of CPN‐IMIP hydrogels: (A) Frequency and (B) temperature sweep analysis of hydrogels, (C) complex modulus of hydrogel samples, (D, E) shear stress and pre‐gelation viscosity of composite hydrogels (T = 25°C), (F) maximum adhesion force  (F_max_) of composite hydrogels, and (G) schematic illustrating interactions between mucin and hydrogels.

According to the results of rheological examination of the CPN‐IMIP hydrogel‐based eye drops in Figure 5A, the storage modulus (G') in every sample at low frequencies continuously outperformed the loss modulus (G″), suggesting that the hydrogel eye drops behaved more like elastic materials than viscous ones. Notably, the G' of all samples started to decrease after 10 rad.s^−1^, crossing G'', showing a viscoelastic behavior in higher frequencies. Moreover, G' was getting closer to G'' as the oscillatory frequency enlarged. All samples followed the same trend, except for CPN‐IMIP35, for which G' and G'' did not cross in the examined range of frequencies and maintained its elastic properties. Moreover, the inclusion of IMIP particles significantly increased both G′ and G″ moduli relative to CPN‐IMIP0, suggesting reduced polymer chain mobility through enhanced intermolecular interactions that reinforced the hydrogel network. This behavior suggested that higher microgel concentrations increase the resistance of hydrogels to shear forces, resulting in the formation of more durable and stable hydrogels [59]. Carrelo et al. [60] observed a similar trend in the increasing storage modulus of samples with higher concentrations of microparticles when investigating a thermoresponsive injectable dual‐release system based on Pluronic hydrogel containing gellan gum/alginate microparticles [60]. The temperature sweep analysis of composite hydrogels was performed to determine the thermal responsiveness of hydrogels, and the results are presented in Figure 5B. Notably, the pure hydrogel exhibited a downward trend in moduli at higher temperatures, suggesting a weakening of the gel structure [61, 62]. Both moduli increased in the sol state due to the presence of microgels. In all composite hydrogels, G' was higher than G″, indicating elastic behavior.

Moreover, the moduli increased sharply at LCST and kept rising with temperature. This trend might be a result of stronger particle‐matrix interactions and interconnected bonds. Similar effects were reported in previous rheological studies [60]. It is worth noticing that the pH‐responsive behavior of this eye drop is mainly attributed to the IMIPs within the hydrogel matrix and affects swelling and drug release rather than the viscoelastic properties of the hydrogels.

The complex modulus (G*) was also a critical factor, indicating the overall resistance to deformation calculated and shown in Figure 5C. It can be observed that the complex modulus of all hydrogels decreased with increasing frequency. This indicated that all samples demonstrate a shear‐thinning behavior [60]. Pre‐gelation viscosity was also evaluated to determine the fluidity and flow characteristics of samples, and the results are presented in Figure 5D,E. Results showed that the viscosity varied from 3.1 to 37.3 mPa.s for CPN‐IMIP10 to CPN‐IMIP35. The viscosity remained constant regardless of the shear rate. This indicated that the fluid exhibited Newtonian behavior, and it flowed consistently under both low or high shear conditions. The Newtonian behavior and increased viscosity at higher particle concentrations. This could be particularly beneficial in eye drops designed to deliver medication over an extended period [52, 63, 64].

### 2.7 Mucoadhesive Properties of Composite Hydrogels

Mucoadhesive properties of hydrogels were examined using fresh cow mucin through an adhesion test, and the results are presented in Figure 5F. As can be seen, F_max_ increased in the presence of mucin, revealing that hydrogel eye drops interacted effectively with mucin [64]. It was also observed that with the increase in IMIP concentration, F_max_ enhanced. The principal interaction of mucin‐composites is shown in Figure 5G. Alginate is an anionic, mucoadhesive polymer and can form a hydrogen bond with mucin‐type glycoprotein through interactions between carboxyl and hydroxyl [65]. Calcium ions, used for crosslinking of alginate, can also interact with the negatively charged sialic acid residues in mucin, enhancing electrostatic interactions. Also, Cs is well recognized for its strong mucoadhesive properties due to its cationic nature. Under physiological conditions, the protonation of chitosan's primary amine groups leads to the formation of electrostatic bonds with negatively charged residues present on the ocular surface [66]. These interactions, together with the effects of alginate and calcium ions, may synergistically enhance adhesion between the hydrogel to mucosal surfaces, thereby increasing mucoadhesive properties [67].

### 2.8 Temperature and pH‐Responsive Properties of Composite Hydrogels

**FIGURE 6:**
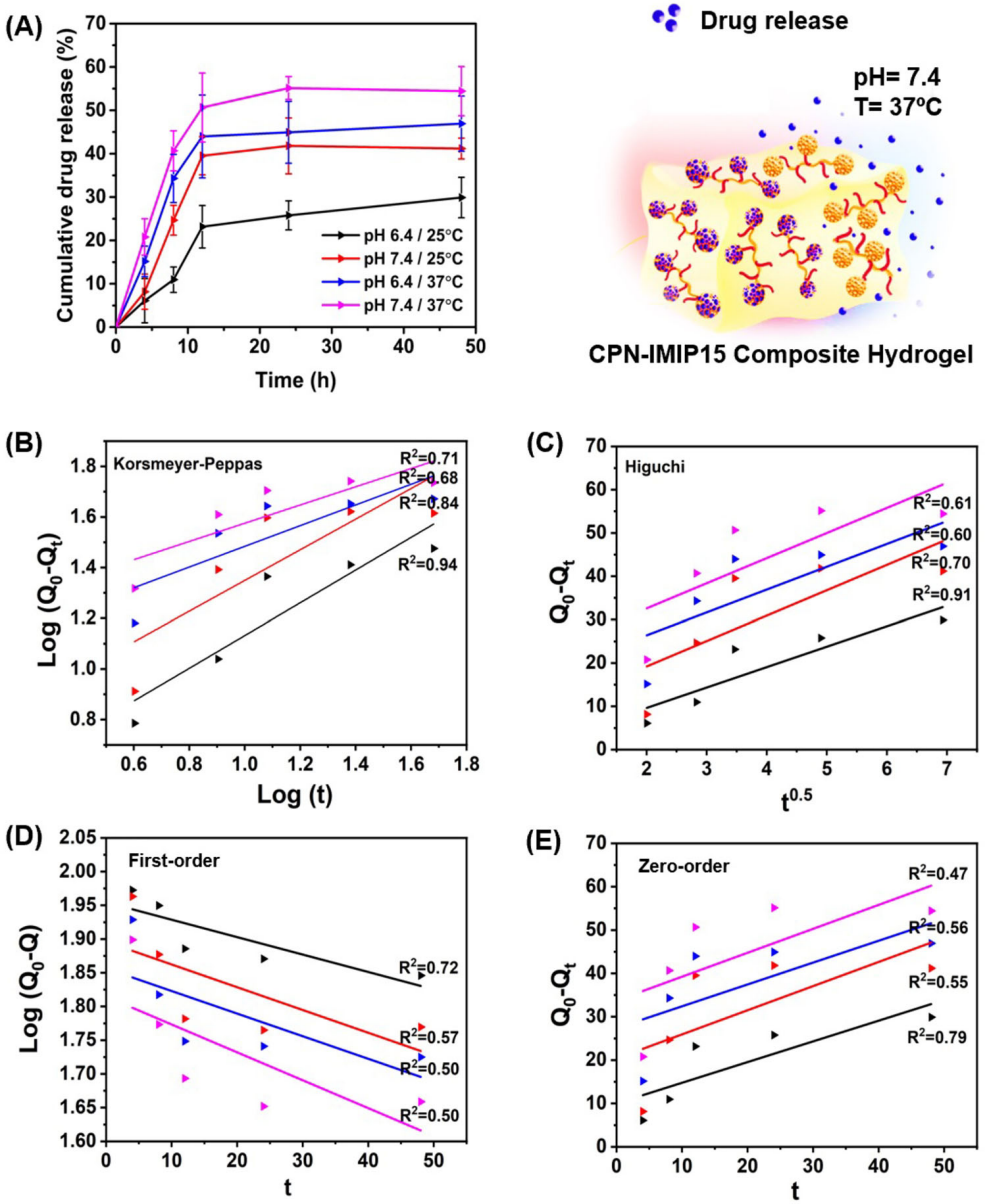
INS release form CPN‐IMIP hydrogels: (A) Cumulative release percentage of INS from composites during 48 h of incubation in STF while considering the variations in two parameters: pH (6.4 and 7.4) and temperature (25°C and 37°C), kinetics of insulin release; (B) the Korsmeyer‐Peppas, (C) Higuchi, (D) first‐order, and (E) zero‐order models.

Dry eye syndrome disrupts tear production and tear film stability, leading to alterations in the physiological pH of the eye. In our previous study, pH‐dependent drug release of IMIPs was investigated [24]. In the present study, INS release of this dual‐responsive hydrogel was examined by changing both pH and temperature of the environment. Among all formulations, CPN‐IMIP15 was selected for pH and temperature sensitivity studies due to its optimal rheological and physiological properties in previous experiments. Figure 6A depicts the cumulative release of INS from CPN‐IMIP15 during 48 h of incubation in STF with different pH values (6.4 and 7.4). The findings demonstrated that all hydrogels had burst release during the first 10 h of incubation *in vitro*, and that the release patterns of INS from CPN‐IMIP0 and CPN‐IMIP15 were nearly similar. Drug diffusion, hydrogel swelling, and degradation are some of the processes that may be responsible for INS release. At pH 6.4, about 29.8 ± 3.6% of INS was released over 48 h at 25°C (below the LCST). Increasing the pH to 7.4 resulted in a higher release of 41.1 ± 2.1% INS at the same temperature. When the temperature was enhanced to 37°C, the release rate increased further: 46.9 ± 1.5% at pH 6.4 and 54.4 ± 4.8% at pH 7.4. The higher release rates in pH 7.4 can be attributed to electrostatic interactions and the pH‐responsive nature of the imprinted microgels.

Additionally, the pH of the surrounding environment influenced INS stability; at pH 7.4, insulin is more soluble and stable, whereas at pH 6.4, it is more prone to accumulation [68]. Moreover, the release rate at 37°C was higher than that at 25°C. This increased rate can be explained by the collapse of PNIPAAm chains, leading to pore formation in the copolymer structure at temperatures above the LCST. As a result, the permeability of hydrogels to water molecules increased, facilitating enhanced INS release [69, 70, 71]. The graphs of INS release kinetics are shown in Figure 6B–E. For all samples during 48 h, the Korsmeyer‐Peppas model (Figure 6B) exhibited the best linear fit, with the highest correlation coefficients (R^2^), followed by the Higuchi model (Figure 6C). The first‐order and zero‐order models (Figure 6D,E), on the other hand, showed poor fit for describing the kinetics of insulin release, as seen by their lower correlation coefficients across all samples. Additionally, the exponent “n” in the Korsmeyer‐Peppas model was evaluated to characterize the INS release mechanism. At 37°C and pH 7.4, the “n” value was higher than 0.43, suggesting a non‐Fickian diffusion process linked to release that is controlled by swelling. A diffusion‐controlled release was indicated by an n value < 0.43 at pH 6.4 and 25°C. At pH 6.4 and 25°C, INS release was mainly diffusion‐controlled. This might be due to the denser hydrogel network below the LCST and lower solubility of INS, resulting in a slower release rate.

### 2.9 Cytocompatibility of Composite Hydrogels

**FIGURE 7:**
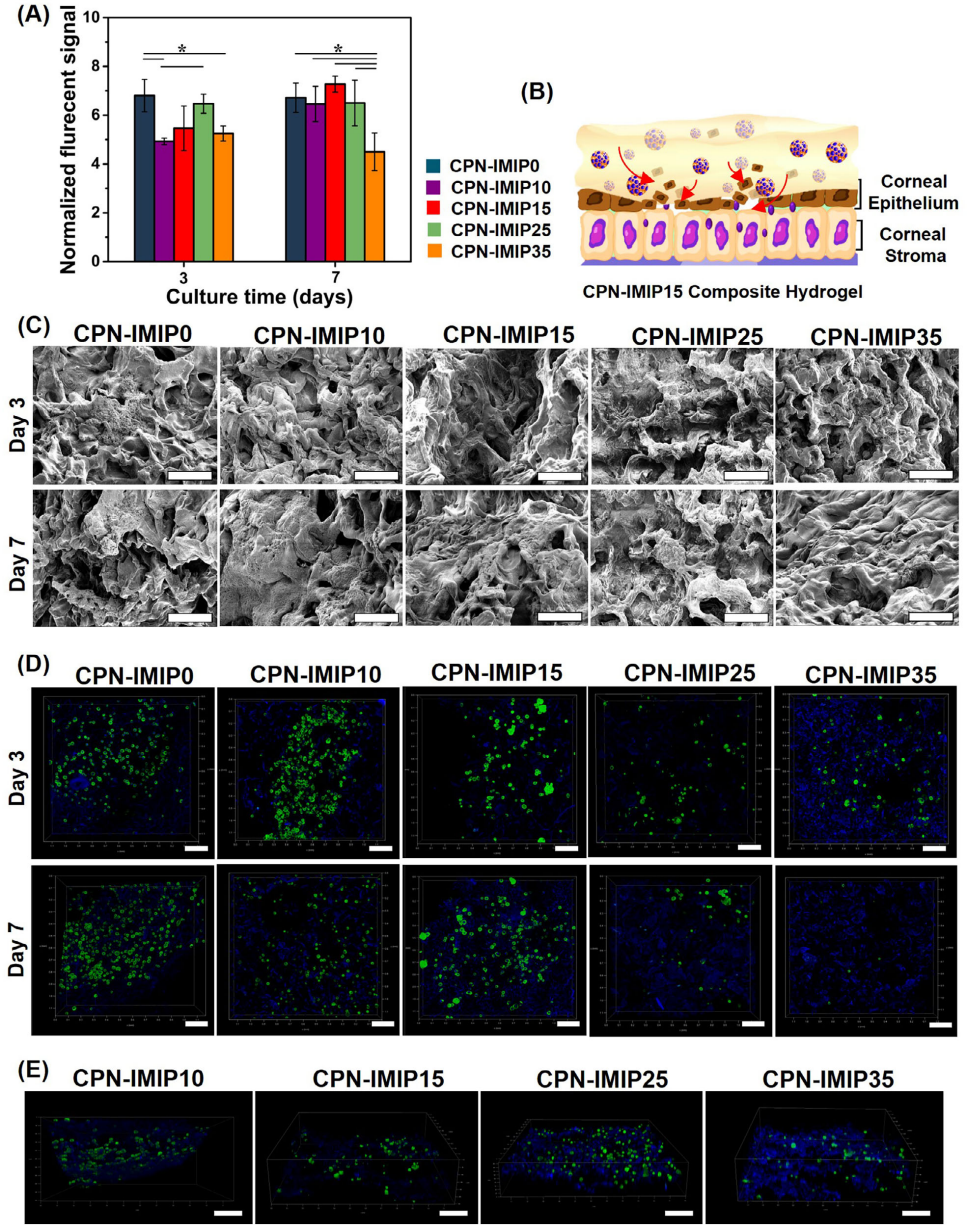
The cytocompatibility of CPN‐IMIP hydrogels against HCECs: (A) Alamar Blue cell viability assay of composite hydrogels against HCECs (3 and 7 days) (n = 3, *: p < 0.05); results are normalized to day 1, (B) schematic of the CPN‐IMIP15 composite hydrogel on the ocular surface showing sustained drug release and cell migration through hydrogel matrix, (C) SEM images (50 µm) of HCECs attached and spread on hydrogel surfaces after 3 and 7 days of culture, (D) surface and (E) cross sectional images taken by confocal laser scanning (scale: 200 µm) from HCECs cultured in CPN‐IMIP hydrogels.

The cytocompatibility of the composite hydrogels was tested using human corneal epithelial cells (HCECs) via Alamar Blue assay (Figure 7A). After 3 days of culture, the highest absorbance was measured for CPN‐IMIP0, offering an optimal environment for cell attachment and growth. This finding was likely due to the hydrophilic nature of the pure Cs‐g‐PNIPAAm hydrogel, which promoted cell adhesion without the structural interference caused by microgels. As microgels were added, cell response varied with concentration; specifically, CPN‐IMIP25, CPN‐IMIP15, CPN‐IMIP35, and CPN‐IMIP10 exhibited the highest normalized absorbance of fluorescence signals, likely due to enhanced surface properties and hydrophilicity. On day 7, all samples generally showed higher metabolic activity, indicating cell proliferation over time. CPN‐IMIP15 showed the greatest increase in metabolic activity over 7 days from 5.6 ± 0.6 to 7.3 ± 0.3, followed by CPN‐IMIP25 and CPN‐IMIP10. The sustained release of INS from the composite hydrogels likely contributed to increased cell viability, as INS is known to support cell survival and metabolic activity [72]. The observed trend correlated with moderate microgel concentrations, which provided an optimal balance of porosity and hydrophilicity. This promoted nutrient and oxygen transport while offering sufficient surface area for cell attachment [73, 74, 75]. By contrast, CPN‐IMIP35 displayed a declining trend in metabolic activity, with values decreasing from 5.2 ± 0.3 on day 3 to 4.5 ± 0.7 on day 7, likely due to the high microgel content and increased insulin concentration in the medium, making the environment toxic and less conducive to sustained cell growth [52, 56]. Furthermore, the degradation of calcium alginate microgels could release acidic byproducts, leading to a localized drop in pH. Such an acidic environment might disrupt ionic homeostasis, impair the extracellular matrix, and create a less favorable environment for cell proliferation [76, 77]. The degradation of these microgels, particularly at higher concentrations, may account for the overall decline in cell growth observed in certain samples. Figure 7B illustrates that the composite hydrogel forms a thin layer on the ocular surface. Its porous structure enables diffusion of cells and helps tissue regeneration. Meanwhile, the sustained drug release leads to accelerating this process. The SEM micrographs from the adherent cells on the surface of the composite hydrogels are presented in Figure 7C. As can be seen, the surface of CPN‐IMIP10 and CPN‐IMIP15 samples is uniformly covered with cells after 7 days of the culture process, while in other samples, the cell growth is in the form of colonies and less homogeneously expanded. The pore structure seen in CPN‐MIP10 and CPN‐IMIP15 likely facilitates the spread and anchorage of cells, providing them with a stable and interconnected surface.

In such an environment, cells began to grow and stay close to each other to form cell‐cell interactions. They overlap within the pore structure, and individual cells were not easily discernible. In contrast, CPN‐IMIP0, CPN‐IMIP25, and CPN‐IMIP35, with either no microgels or excessive particle concentration, likely created uneven or dense surfaces, leading to isolated colonies of cells rather than uniform growth. We further examined the cytoskeleton of HECEs cultured on hydrogel samples using phalloidin staining and imaged them using a confocal microscope, revealing the nucleus of cells in blue and the cytoskeleton in green. Figure 7D represents the spreading cells after a culture period of 3 and 7 days. The confocal images revealed highly proliferative cells covering the surface after 7 days of culture, especially on CPN‐MIP15 films. The cross‐sectional confocal images (Figure 7E) indicated that cells had effectively penetrated and diffused into the scaffold structure, showing deep integration within the hydrogels. Wiltsey et al. [78] reported the development of an injectable PNIPAAm‐based scaffold containing 50 mg.mL^−1^ alginate microparticles. They showed that the incorporation of microparticles enhanced tissue adhesion and maintained the survival of the epithelial‐like cells [78]. This highlights the biocompatibility of the hydrogel, as cells were able to thrive not just on the surface but within the internal matrix, maintaining their viability and division throughout the depth of the material [29, 79]. This penetration and sustained biocompatibility suggested that the hydrogels provided a suitable environment for cellular activities such as adhesion, proliferation, and migration. For DES, where epithelial damage or regeneration is a concern, a hydrogel that supports cellular integration and division is critical. The ability of cells to penetrate and maintain biocompatibility within the scaffold suggested that these hydrogels can serve as a protective and regenerative layer, promoting healing in the damaged corneal epithelium [79]. In research by Mellati et al. [79], a similar cell behavior was observed in a PNIPAAm‐based hydrogel as a microenvironment for the growth of stem cells. In this study, the analysis of confocal images confirmed that the cells were highly viable, penetrated uniformly into different depths of the hydrogel, and created cell biomass inside the hydrogel. Their results confirmed that this hydrogel can provide a potential 3D microenvironment for stem cell culture and differentiation [79].

## 3 Animal Studies in Rat Models With DES Syndrome

**FIGURE 8:**
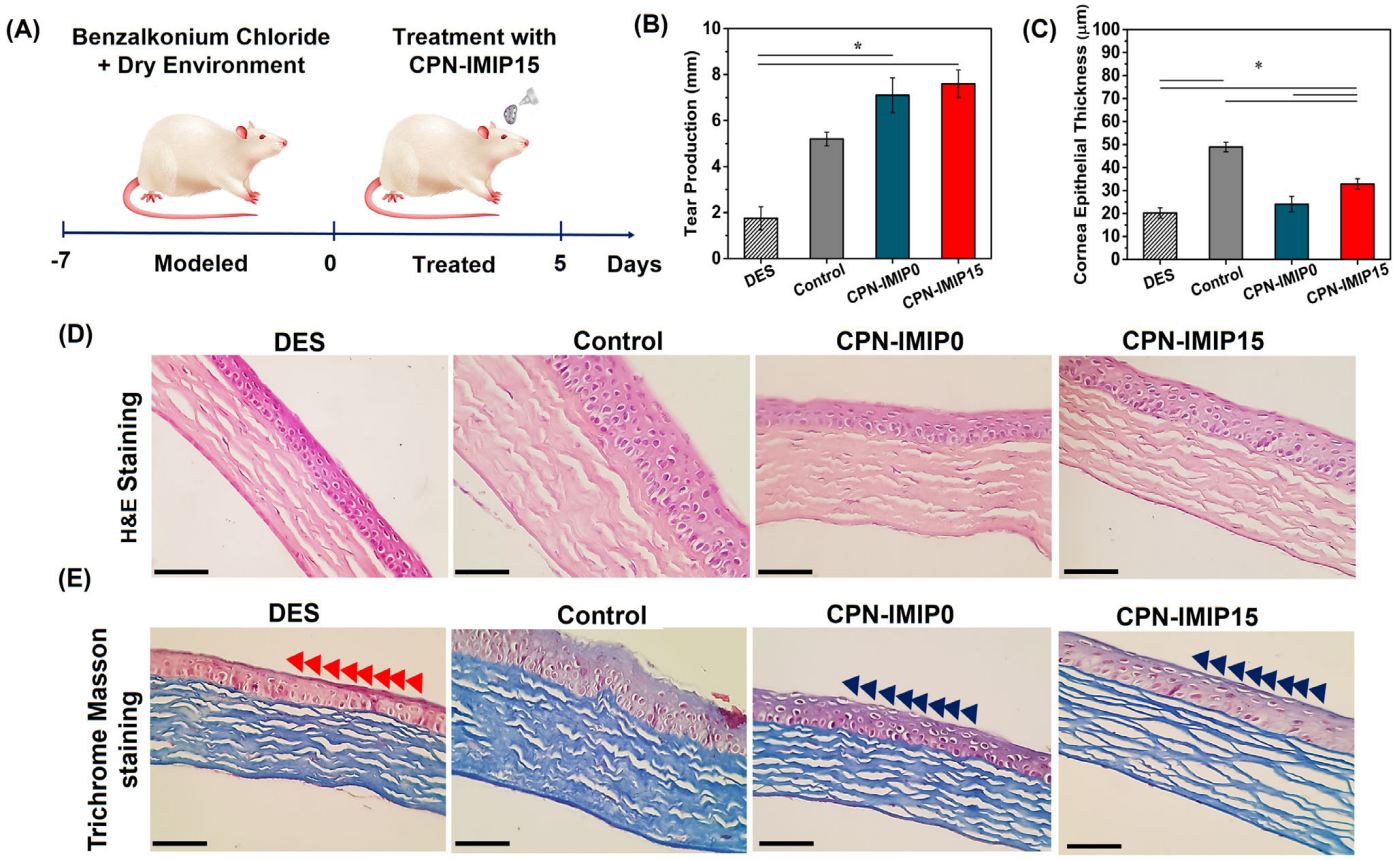
*In vivo* results of CPN‐IMIP hydrogels: (A) experimental timeline, (B) the wetted length of the Schirmer paper strip for the corneas with experimentally induced dry eye syndrome (DES), control, and treated for 5 days after topical administration of CPN‐IMIP0 and CPN‐IMIP15 solutions, (C) thickness values, and (D) histological images of DES, control, and treated for 5 days after topical administration of CPN‐IMIP0 and CPN‐IMIP15 solutions (n = 3, *: p < 0.05), and (E) collagen formation evaluation with Masson trichrome staining; Scale bar = 100 µm.

Since tears shield the corneal epithelial cells from the ocular surface's dryness, tear production is a crucial clinical indicator for assessing the formation of dry eye [67]. To further investigate the impact of the composite hydrogels in the rat model with DES syndrome, we treated animals with the CPN‐IMIP15 formulation and monitored the tissue behavior. Figure 8A shows the timeline of the performed animal test. Over 7 days, animal models with DES syndrome were created by the administration of benzalkonium chloride (BAC), leading to partial disruption of the mucin layer [80]. After this step, we treated a group of 3 animals with CPN‐IMIP15 eye drops for 5 continuous days twice a day. According to the results, topical application of CPN‐IMIP15 significantly increased tear production as compared with the other group (Figure 8B). Based on these findings, CPN‐IMIP15 was a formulation that effectively reversed the deficient production of tears caused by DES. Figure 8C displays the corneal epithelial thickness determinations based on Hematoxylin and Eosin (H&E) staining images. In the control group, the thickness of the epithelial layer was 48.2 ± 2.1 µm, which followed the reported value for normal rat corneal epithelium [81]. The corneal epithelial layer was considerably thinner (p < 0.05) following BAC administration. Further increase in epithelial cell loss was shown by the considerably reduced epithelial layer thickness in the DES groups (20.2 ± 3.2 µm) compared to the control and treatment groups (24 ± 3 µm and 32 ± 2 µm for CPN‐IMIP0 and CPN‐IMIP15, respectively) (p < 0.05). According to Figure 8D, after 5 days, the CPN‐IMIP0 group showed partial epithelial regeneration with improved thickness but less cellular uniformity and organization compared to the control. Notably, at the same period, the CPN‐IMIP15 group demonstrated significant epithelial restoration with a well‐defined, stratified structure.

The thermoresponsive behavior, which led to increased viscosity of the formulation at the ocular surface temperature, together with the electrostatic interactions with residual mucin, could result in prolonged retention of the hydrogel on the ocular surface and lead to the observed increase in tear production and epithelial thickness. This trend was also evident in similar previous studies [82, 83]. Collagen deposition is also a major problem throughout the DES healing process. To assess collagen deposition in the corneal tissue, Masson's trichrome staining was used. According to the results, the appearance of blue color on the epithelial sites after treatment with INS‐loaded hydrogels clearly showed histological evidence of corneal tissue healing. (Figure 8E). In the CPN‐IMIP0 group, moderate recovery of epithelial thickness was observed. In contrast, the CPN‐IMIP15 group showed a more continuous and stratified epithelial layer and regular collagen bundles, closely resembling the control. These results suggested that CPN‐IMIP15 formulation enhanced insulin stability and provided a more sustained release, leading to superior tissue regeneration and extracellular matrix remodeling compared to non‐encapsulated insulin delivery (CPN‐IMIP0).

## 4 Conclusions

This study focused on developing a drug delivery system combining surface‐imprinted calcium alginate microgels and Cs‐g‐PNIPAAm hydrogel in the form of an eye drop. To modulate the physical, mechanical, and biological characteristics, IMIP particle concentrations were varied between 10 and 35 mg.mL^−1^ during the fabrication process. The resulting CPN‐IMIP composite hydrogel demonstrated three key advantages over Cs‐g‐PNIPAAm hydrogel: (1) reduced degradation kinetics, (2) decreased swelling behavior, and (3) enhanced rheological properties at LCST and above. The most suitable concentration of microgels was found to be 15 mg.mL^−1^using morphological, biological, and rheological characterization. The introduction of 15 mg.mL^−1^ microgels to the hydrogels decreased the sol–gel transition temperature to 31.1± 0.5°C, ideal for ophthalmic drug delivery systems. Moreover, the microgel presence, within the studied concentrations, altered the rheological modulus of Cs‐g‐PNIPAAm hydrogel and led to the creation of a more mechanically stable structure. The CPN‐IMIP15 composite hydrogel released INS within 48 h, in response to pH and temperature change, making it possible for INS to be released in a dual‐responsive manner. Cytotoxicity analysis also revealed that CPN‐IMIP15 could successfully support HCECs’ proliferation and growth. *In vivo* results based on a dry eye model in rats also indicated improved tear secretion, increased epithelial thickness, and collagen formation on the cornea epithelial site when treated with CPN‐IMP15. In conclusion, with its controlled release, excellent biodegradability, low swelling rate, and suitable rheological behavior, CPN‐IMIP15 could be a promising drug delivery system for the treatment of DES.

## 5 Experimental Section

### 5.1 Materials

Sodium alginate (SA, Mw = 80,000–120,000 Da) was obtained from Sigma‐Aldrich Co., USA. Calcium chloride, sodium chloride, isopropanol, Tween‐80, span‐80, liquid paraffin, and acetic acid (C_2_H_4_O_2_) were from Merck Co., USA. N‐isopropyl acrylamide (NIPAAM), Chitosan (Cs, Mw = 1.4–2.2×10^5^ Da), N, N‐methylene bisacrylamide (MBA), sodium dodecyl sulfate (SDS), potassium persulfate (KPS), and glacial acetic acid were also purchased from Sigma‐Aldrich Co., USA. For cell culture experiments, reagents such as formaldehyde, apo‐transferrin, epinephrine, hydrocortisone hemisuccinate, Extract P were purchased from Merck, USA. Cornea epithelial (CE) growth factor, L‐glutamine, phenol red, trypsin‐EDTA, and penicillin‐streptomycin‐amphotericin were purchased from ATCC. Alamar blue reagent was purchased from Sigma‐Aldrich, USA. Moreover, Alexa Fluor 488 Phalloidin (Invitrogen), 4′,6‐diamidino‐2‐phenylindole (DAPI, Sigma Aldrich), Triton‐X solution (Merck, USA), and bovine serum albumin (BSA, Merck, USA) were also purchased for cell staining assays. Dialysis membrane (13‐14 kDa) was provided by Betagen Co., Iran. Sodium chloride (NaCl), simulated tear fluid (STF), and human insulin (INS, Lansulins R, 100IU per 1 mL) were purchased from Exir Pharmaceutical Co., Iran. Moreover, deionized distilled water (DDW) was used for experiments.

### 5.2 Synthesis of Cs‐g‐PNIPAAm Copolymer

The Cs‐g‐PNIPAAm copolymer was synthesized through a free radical polymerization using a method established by Zheng et al. [84]. Concisely, Cs (3 g) was fully dissolved in 180 mL of a 1 wt.% acetic acid solution inside a three‐necked flask. Subsequently, a solution containing NIPAAM (6 g), MBA (0.12 g), and SDS (0.06 g) was prepared in 60 mL of DDW and added to the Cs solution. Following agitation under a nitrogen atmosphere, the temperature of the system was enhanced to 70°C, and after 3 h, 0.026 g dissolved KPS in 15 mL DDW was added to the solution. The reaction was further kept under a nitrogen atmosphere at 70°C for 5 h to remove oxygen and prevent any interaction with free radicals. After polymerization, the copolymer solution was dialyzed at 25°C in DDW for 120 h to remove unreacted monomers. During this process, dialysis water was changed once every 24 h. After dialysis, the copolymer solution was subjected to 24 h of freeze‐drying and then stored at 4°C for further use.

### 5.3 Synthesis of IMIPs

The MIP particles were synthesized based on the emulsification‐gelation procedure employed in our previous publication [24]. Briefly, a 2 wt.% aqueous sodium alginate (SA) containing 1 mg.mL^−1^ insulin was added dropwise to the oil phase containing liquid paraffin (9.6 mL), Span‐80 (0.3 mL), and Tween‐80 (0.1 mL) at room temperature (RT). Afterward, a 5 wt.% solution of calcium chloride (1.2 mL) was added dropwise to the resulting mixture. The mixture was then agitated for 3 h at 400 rpm. To ensure the full precipitation of the microgels, the solution was kept undisturbed overnight. Precipitated microgels were washed three times with isopropanol and water using centrifugation at 3500 rpm for 10 min to remove any loosely bound or free INS. Then, the precipitated microgels were subjected to freeze‐drying for a duration of 24 h to eliminate any residual moisture. To remove insulin from microgels and obtain MIPs, microgels were washed with 0.01 M PBS (pH = 7.4) at RT. The elimination of insulin from the supernatant following each rinse was assessed using a UV‐Vis spectrophotometer (Onlab, EU‐2800 DS, China) set to λ_max_ = 276 nm. Until no insulin was found in the solution, this washing process was repeated. 5 mg of MIPs were immersed in a solution containing insulin at a concentration of 5 mg.mL^−1^ in PBS at 25°C and gently stirred for 2 h in order to load insulin into empty microgels. Following centrifugation of the resultant mixture with water and isopropanol to remove free insulin, the IMIPs were freeze‐dried overnight and stored at 4°C. It should be noted that insulin incorporated during the first step served as a template to create bonding sites within the polymer matrix. After template removal, the second loading step was performed to create the final IMIPs. According to our previous publication, at optimized concentration, the loading effectiveness of INS in MIPs was around 80 ± 3% [24].

### 5.4 Fabrication of Composite Hydrogels

Composite hydrogels based on Cs‐g‐PNIPAAm and containing IMIPs were synthesized via physical blending, following the methodology outlined by Wiltsey et al. [78]. First, a solution containing 5 wt.% Cs‐g‐PNIPAAm (1 mL) was prepared in 0.01 M PBS. Then, different concentrations of IMIPs (10, 15, 25, and 35 mg.mL^−1^) were added to the copolymer solution, thoroughly blended at RT for 1 h, and then the temperature was raised to 37°C for complete gelation. The resulting soft hydrogels were labeled as CPN‐IMIP0, CPN‐IMIP10, CPN‐IMIP15, CPN‐IMIP25, and CPN‐IMIP35 according to the IMIP concentration (0, 10, 15, 25, and 35 mg.mL^−1^) in the final composites. Subsequently, the samples were lyophilized for 24 h and used for further characterization.

### 5.5 Structural and Chemical Characterization

The structural features, pore size of the samples, and IMIP diameters were analyzed using an SEM (Philips‐XL30). Before imaging, the samples were sputter‐coated with gold for 120 s. Moreover, atomic force microscopy (AFM, Bruker, Germany) was used to map the surface topography of MIP and IMIP. Using an intermittent technique at RT, the studies were carried out in contact mode with a resonance frequency between 300 and 100 kHz. A scanning line rate of 0.2 to 0.5 Hz was used to scan and analyze the samples. Additionally, using a Horiba SZ‐100 (Japan), zeta potential measurements of diluted IMIP and MIP were carried out in PBS at pH 7.4 and 25°C. Additionally, to evaluate the effectiveness of the copolymerization, the grafting parameters, such as total monomer conversion (X%), grafting efficiency (GE%), and grafting ratio (GR%), were determined in the dry state for all composite hydrogels using the following equations [85]:

(1) $$X\%=\left(\left(W_{f}-W_{Cs}-W_{APS}\right)/\left(W_{m}+W_{MBA}\right)\right)\times 100$$

(2) $$GR\%=\left(\left(W_{f}-W_{Cs}\right)/W_{Cs}\right)\times 100$$

(3) $$\mathrm{GE}\%=\left(\left(W_{f}-W_{\mathrm{Cs}}\right)/W_{m}\right)\times 100\;$$

where W_Cs,_ W_APS,_ W_MBA_, W_f,_ and W_m_ are the weights of chitosan, APS, MBA, the final product, and NIPAAm monomer, respectively. To assess the chemical structure of the copolymer and the formation of the composite, FTIR spectroscopy (Tensor 27, Bruker) was utilized from 400 to 4000 cm^−1^. Differential scanning calorimetry (DSC, Metler Toledo) was used to determine the samples' phase transition temperatures. For this analysis, samples were placed in an aluminum pan and heated from 20°C to 300°C at a rate of 10°C.min^−1^ under a nitrogen atmosphere. Furthermore, thermal gravimetric analysis (TGA, Metler Toledo) was conducted on the samples. A platinum container containing 8–10 mg of each sample was heated at a heating rate of 10°C.min^−1^ from 20 to 600°C under a nitrogen atmosphere, purged in the system at a flow rate of 40 mL.min^−1^.

### 5.6 pH Measurements

A pH meter (Metrohm 827, Switzerland) was used to measure the pH of composite samples at RT. Three separate pH tests were performed on each formulation, and the mean ± SD value was determined.

### 5.7 Light Transmittance Determination

According to earlier research, hydrogels are deemed transparent if their light transmittance in the visible wavelength range of λ = 390–780 nm is greater than 90%. Translucent hydrogels have a light transmittance between 10% and 90%, and opaque hydrogels have a light transmittance of less than 10%. Hydrogels were swollen in DDW at 25°C, and a UV‐Vis spectrophotometer was used to evaluate the composite hydrogels' optical transparency [86]. To accomplish this, the hydrogels were added to 1 mm‐thickness cuvettes. At λ = 650 nm, the light transmission was then measured. Equation (4) was used to determine the hydrogel's light transmittance:

(4) A=−LogT%

where A is absorbance, and T% is light transmittance.

### 5.8 Protein Adsorption Measurements

In ocular formulations, surface protein adsorption is considered crucial, as protein accumulation is directly related to inflammation and microbial attachment. Therefore, protein adsorption measurements were performed to evaluate the ability of the hydrogel matrix to adsorb serum proteins. The hydrogel samples were soaked in 10 mL of a 100 mg.L^−1^ BSA solution in PBS after being swollen in PBS for 2 h. After incubation for 12 h at 37°C and 120 rpm in a shaker incubator, the hydrogel was rinsed with fresh PBS to remove any loosely adhered proteins. A UV‐Vis spectrophotometer was used to measure the amount of desorbed protein at λ_max_ = 278 nm [87]. Notably, all formulations were tested under identical experimental conditions, and the presence of any residual or released insulin at the absorbance wavelength of 278 nm is considered to be minimal compared to the BSA concentration used in the protein adsorption study.

### 5.9 Physiological Stability Evaluation

Using a water contact angle measurement apparatus (CA‐500A, Iran), the static water contact angle was measured to evaluate the hydrophilicity of the composite hydrogels. In this regard, lyophilized hydrogels were swollen for 1 h using DDW. The fully swollen hydrogels (n = 3) were blotted with a filter paper to remove free water. A 5 µL drop of distilled water was placed on the surface of the samples, and the Dino‐Lite camera captured an image of the droplet after 3 s. Furthermore, to evaluate the effect of MIP concentration on the swelling rate of the composite hydrogels, samples (n = 3) were first weighed (W_1_) and then immersed in STF solution (pH 7.4) at 37°C for 1 h. Next, the weight of the swollen samples was measured (W_2_), and the swelling ratio (SR) was determined according to Equation (5) [73]:

(5) $$Swelling\;ratio\left(\%\right)=\left(W_{2}-W_{1}\right)/W_{1}\times 100$$

where W_1_ is a dry weight, and W_2_ is a weight after lyophilization and weighing. Moreover, the physiological stability of the samples was also examined. First, the dry weight (W_1_) of the hydrogels (n = 3) was recorded. The hydrogels were then placed in STF (pH 7.4) at 37°C, with the PBS solution renewed every 24 h. After immersion for 1, 3, 5, and 7 days, the samples were lyophilized, weighed (W_2_), and their weight loss was calculated using Equation (6) [73]:

(6) $$\mathrm{Weig}\mathrm{ht}\;\mathrm{loss}\;\left(\%\right)=\left(W_{1}-W_{2}\right)/W_{1}\times 100$$

### 5.10 Ex Vivo Mucoadhesive Strength Measurements

The Ex vivo adhesive forces of the hydrogels were measured using a texture analyzer, using a 5 kg load cell provided with a 10 mm cylindrical analytical probe, in order to determine the maximum detachment force (F_max_). Fresh cow mucin was obtained from a local slaughterhouse and used immediately. A 5 wt.% cow mucin dispersion was made in DDW. Filter paper was used to create mucin films. On the filter paper, the 300 µL mucin dispersion was evenly distributed. The mucin dispersion was allowed to dry by heating the filter sheets at 70°C for 5 min, after which the sheets were fixed to the stationary platform of the texture analyzer. On the other side, the filter paper was carefully covered with 0.5 mL of swollen composite hydrogels with various concentrations of IMIP. The composite hydrogels were subjected to a force of 0.3 N for a minute, after which the upper plate was raised steadily at a speed of 0.5 mm.s^−1^ until the hydrogels completely detached. Three replicate analyses were performed for each formulation, each using a different sample and mucin film. The software recorded the force of detachment as a function of displacement, and the results were compared with the adhesion strength study data without a mucin film. The difference between the two forces with and without mucin (F_max_) represents the hydrogel's mucoadhesion capacity. Measurements were performed in triplicate, and the results were reported as mean ± SD (n = 3).

### 5.11 Rheological Measurements

Rheological evaluations were performed to determine the flow behavior and viscoelastic properties of hydrogel formulations, which were critical for the topical administration and ocular retention. A proper pre‐gelation viscosity allows ease of administration into the eye as a liquid, and it's a main prerequisite for an eye drop. Therefore, the flowability of the hydrogel eye drops was evaluated using a rheometer (AR2000, TA Instruments, USA). Using a cone‐plate geometry (plate diameter: 25 mm, gap: 3 mm, 2° angle) and varying the shear rate from 1 to 10 s^−1^, the viscosity of each formulation was determined at RT. The frequency sweep was from 0.1 to 100 rad.s^−1^, and oscillatory measurements were made at 1 Hz for the temperature sweep measurements, while the temperature was raised at a rate of 3°C.min^−1^ between 25°C and 60°C. As a result, the dynamic viscoelastic functions, such as the dynamic shear storage modulus (G') and loss modulus (G″), were calculated as a function of temperature and angular frequency.

### 5.12 In Vitro INS Release From Composite Hydrogels

The pH‐ and temperature sensitivity of composite hydrogels was investigated to evaluate the release behavior of the samples. Samples (n = 3) were immersed in STF and incubated at various temperatures (37°C and 25°C) and pH levels (7.4 and 6.4). At the specific time points (4, 8, 12, 24, and 48 h), 3 mL of supernatant was collected, and the absorbance was measured using a UV‐Vis spectrophotometer at λ = 276 nm. Finally, the released INS was calculated using an INS standard curve and Equation (7) [88]:

(7) $$\mathrm{Cumu}\mathrm{lati}\mathrm{ve}\;\mathrm{insu}\mathrm{lin}\;\mathrm{rele}\mathrm{ase}\;\left(\%\right)=M_{t}/M_{\infty }\;\times 100$$

where M_t_ is the amount of INS released at time t, and M_0_ is the initially loaded insulin.

To determine the kinetic model of INS release, various models, the zero‐order model (Equation 8), first‐order model (Equation 9), the Higuchi model (Equation 10), and the Korsmeyer‐Peppas model (Equation 11) were employed to choose the equation that best fits the data and to examine the mechanism of insulin release from CPN‐MIP15 hydrogel [89].

(8) $$M_{t}/\;M_{\infty }=K\;t$$

(9) $$M_{t}/\;M_{\infty }=1-e^{-\mathrm{Kt}}$$

(10) $$M_{t}/\;M_{\infty }=K\;t^{1/2}$$

(11) $$M_{t}/\;M_{\infty }=K\;t^{n}$$

here, M_t_ is the cumulative amount of INS released at a specific time, M_∞_ is the total INS content loaded into the composite hydrogel, n denotes the release exponent, and t refers to the release duration. The release rate constant for the Zero‐order, First‐order, Higuchi, and Korsmeyer–Peppas models is indicated by K.

### 5.13 In Vitro Cell Culture Studies

To assess the cellular interaction between composite hydrogels and the ocular surface, primary corneal epithelial cells, normal human (HCECs), were purchased from ATCC‐PCS‐700‐010. Cells were cultured using the ATCC corneal epithelial cell growth kit containing a basal medium (BM), supplemented with 5 µg.mL^−1^ apo‐transferrin, 1 µM epinephrine, 100 ng.mL^−1^ hydrocortisone hemisuccinate, 6 mM L‐glutamine, 0.4% Extract P, 5 µg.mL^−1^ insulin, 1 mL of Corneal Epithelial (CE) growth factor, 33 µM phenol red, and 0.5 mL of penicillin‐streptomycin‐amphotericin. Trypsin‐EDTA was used for detaching the primary cells at 80% confluency, followed by applying the Trypsin neutralizing solutions for 3 min. Before cell seeding, the hydrogels with a 5 mm thickness were rinsed with PBS, followed by the sterilization of the samples under UV radiation for 20 min. The cells were counted and seeded with a density of 10^4^ cells/well on the composite hydrogels with different IMIP concentrations and a tissue culture plate (TCP, control) and incubated for 7 days at 37°C and 5% CO_2_. The culture medium was refreshed once a day, and the following experiments were performed.

#### 5.13.1 Cytotoxicity Evaluation

The Alamar blue assay was used to assess the metabolic activity of the composite hydrogels with varying IMIP concentrations on days 1, 3, and 7 of culture. The cell‐seeded films were incubated with a 10 wt.% Alamar blue reagent in a culture medium for 3 h at 37°C. Subsequently, 100 µL of the solution was transferred to a 96‐well plate, and the absorbance was measured at 570–600 nm using a microplate reader (Mithras LB 940, Germany). The results were normalized and reported as mean ± SD (n = 3).

#### 5.13.2 Cell Morphology and Proliferation Evaluation

To examine the impact of varying concentrations of IMIP in composite hydrogels on the growth rate and proliferation of cells, cell‐seeded samples according to the procedure mentioned in section 5.13 were analyzed using a fluorescent microscope after staining of the cells for their cytoskeleton and nucleus. After time points of 1, 3, and 7 days of culture, cells were fixed using a 3.7% paraformaldehyde solution for 15 min. Subsequently, a 0.1% Triton‐X solution was used to permeabilize the cells. Following 5 min incubation, a solution containing 0.2% BSA was introduced to the sample to block aldehyde groups. Then, the cytoskeleton was stained using a diluted Alexa Fluor 488 Phalloidin solution (1:4) in the samples and allowed to incubate for 30 min. Next, a diluted DAPI solution (1:1000) was employed to stain the cell nuclei. Following 5 min incubation in the dark, the samples were rinsed and imaged using a confocal laser scanning microscope (Leica DMI 8 equipped with lasers using LAS X software).

Furthermore, the morphology of fixed cells was analyzed using an SEM (Carl Zeiss Microscopy GmbH, Apreo VS, Thermo Fisher Scientific, Germany). Therefore, the samples were dehydrated using ethanol dilution series (30%, 50%, 70%, 90%, and 97%), 10 min for each, frozen at −80°C with 100% tert‐butanol for 1 h, and lyophilized for 24 h. The samples were sputter‐coated (EM ACE600, LEICA) for 30 s at 30 mA with platinum with a 2 nm thickness before imaging.

### 5.14 Animal Dry Eye Model

To establish a DES model, rats (weighing 250 g; aged 10 weeks) were purchased from the Animal Center of the University of Isfahan (Isfahan, Iran) for implantation of hydrogels on the ocular surface. During the study, the rats were kept in separate cages with free access to water and a regular rat chow diet, in well‐ventilated conditions with temperature‐controlled cages (23–25°C) and a humidity of 30 ± 3% under a 12:12 light‐dark cycle. All animal procedures were authorized by the experimental animal ethics committee (Code of Ethics: IR.UIT.AEC.1403.002) and carried out in accordance with the University of Isfahan's Guidelines for the Care and Use of Laboratory Animals. A DES model was established as previously described [16]. Briefly, animals were treated twice daily for 7 days by topical application of 0.2 mg.mL^−1^ of benzalkonium chloride solution to cause DES in all animals (except in the control group with healthy eyes). After 7 days of DES induction, the rats were randomly divided into 4 treatment groups (n = 3): Control (Group 1), Untreated (Group 2), CPN‐IMIP0 treated (Group 3), and CPN‐IMIP15 treated (Group 4). For 5 continuous days, twice daily, rats were treated by topical application of 5 µL of different eye drop formulations. After 5 days, the rats were euthanized by administering an overdose of chloral hydrate, and the cornea was fixed in 10% formalin and subsequently embedded in paraffin for pathological slide preparation. Histological analysis was then performed as described in 5.14.2.

#### 5.14.1 Tear Secretion Test

After 5 days of administration, the Schirmer test was used to assess the amount of tears produced. Each Schirmer tear test strip was placed into the exterior third of the rat's lower eyelid during the examination without the use of a topical anesthetic. The wetted length of the strip was measured in millimeters after 3 min, and this measurement served as the test result.

#### 5.14.2 Histological Staining

The rat's corneas were removed, collected, and infiltrated with paraffin after being preserved in 4% formaldehyde for at least 24 h. To assess corneal inflammation and collagen formation, paraffinized samples were divided into sagittal slices (5–µm thick), put on glass slides, and stained with H&E and Masson trichrome staining, respectively. For H&E analysis, thin tissue sections were treated with hematoxylin for 3–8 min and 0.5% eosin for 6 min at RT. Following dehydration with alcohol (80% ethanol for 30 s, 95% ethanol for 1 min, 95% ethanol for 1 min, and absolute ethanol for 3 min), the H&E‐stained sections were imaged using a light microscope (40X, Olympus, Japan). TA series of treatments using Weigert's iron hematoxylin solution, Biebrich scarlet acid fuchsin solution, and aniline blue solution were used in the staining procedure. Finally, the stained samples were mounted for further analysis.

### 5.15 Statistical Analysis

A minimum of 3 independent experiments was used to assess and report all numerical results as mean ± standard deviation (SD). Statistical assessments were conducted using GraphPad Prism 7.0 software (La Jolla, CA, USA) via a one‐way ANOVA followed by Tukey's post hoc test for multiple comparisons. The data were considered to be significantly different at p < 0.05.

## Conflicts of Interest

The authors declare no conflicts of interest.

## Data Availability

The data that support the findings of this study are available from the corresponding author upon reasonable request.
